# Contrasting contribution of resident and repopulated brain macrophages in sustaining sleep-wake circuitry

**DOI:** 10.1038/s42003-025-08781-7

**Published:** 2025-09-09

**Authors:** Ali Seifinejad, Mojtaba Bandarabadi, Meriem Haddar, Saskia Wundt, Mehdi Tafti, Anne Vassalli, Abbas Khani, Gianni Monaco

**Affiliations:** 1 Institute of Neuropathology, Faculty of Medicine, University of Freiburg, Freiburg, Germany; 2 Department of Biomedical Sciences, University of Lausanne, Lausanne, Switzerland; 3 Institute of Neuroinformatics, Federal Institute of Technology (ETH) Zurich, Zurich, Switzerland; 4 Present Address: Department of Biomedical Sciences, University of Lausanne, Lausanne, Switzerland

**Keywords:** Neuroimmunology, Glial biology

## Abstract

Sleep is a complex behavior regulated by various brain cell types. However, the roles of brain-resident macrophages, including microglia and CNS-associated macrophages (CAMs), particularly those derived postnatally, in sleep regulation remain poorly understood. Here, we investigated the effects of resident (embryo-derived) and repopulated (postnatally derived) brain-resident macrophages on the regulation of vigilance states in mice. We found that depletion in resident brain macrophages caused increased sleep in the active period, but reduced its quality, reflected in reduced power of brain sleep oscillations. This was observed both for the Non-REM and REM sleep stages. Subsequent repopulation by postnatal brain macrophages resulted in altered, but not fully restored, sleep-wake patterns and additionally induced sleep fragmentation. Furthermore, brain macrophage depletion caused excitatory-inhibitory synaptic imbalance, which was resistant to repopulation, and led to increased inhibitory synapses. At the metabolite level, the distinct metabolite profile induced by brain macrophage depletion largely returned to normal after repopulation. Our findings suggest a so far largely unknown interaction between brain-resident macrophages and sleep and highlight functional differences between resident and postnatally-derived repopulated brain macrophages, paving the way to future exploration of the role of brain macrophages of different origin in sleep disorders and synaptic connectivity.

## Abstract (web summary)

Brain-resident macrophages regulate sleep; their depletion disrupts sleep patterns, synaptic and neurochemical balance. Repopulation partially restores sleep but induces fragmentation and synaptic imbalance.

## Introduction

Sleep is a complex behavior essential for the normal functioning of animals, consistently linked to various diseases when compromised^1^. Sleep regulation involves circadian and homeostatic processes, determining respectively sleep timing and sleep need^2^.

Sleep oscillatory patterns rely on the intricate connectivity of the central nervous system (CNS) at synaptic and neurotransmitter levels. Various cell types, primarily neuronal populations located in the hypothalamus, brainstem (BS), and basal forebrain (BF), interact to finely regulate sleep^3–5^. The expression of synaptic proteins increases during wakefulness and sleep loss, and decreases during sleep^6^, and the postsynaptic protein HOMER1A is a well-established core brain molecular correlate of sleep loss^7^. Additionally, the release of key neuropeptides and neurotransmitters such as HCRT (also called Orexin), serotonin (5-HT), noradrenaline (NA), and acetylcholine across different brain regions actively modulates sleep-wake cycles^8^. This intricate interplay manifests in electroencephalographically (EEG) measurable readouts distinguishing wakefulness, non-rapid eye movement sleep (NREMS), and rapid eye movement sleep (REMS), characterized by distinct brain oscillatory activities such as delta waves (0.5–4 Hz) for NREMS and theta waves (6–9 Hz) for REMS in rodents. However, the contribution of non-neuronal cell types, such as brain-resident macrophages, to brain oscillations and sleep architecture remains largely unexplored.

Brain-resident macrophages comprising parenchymal microglia and CAMs (CNS-associated macrophages)^9–11^, play significant roles in pathology and protecting the CNS from damage^12^. They participate in a range of physiological functions, including synaptic pruning, neurogenesis, immune surveillance^13–15^, and are derived from prenatal sources known as erythromyeloid precursors that engraft into the developing CNS^16,17^, facilitating the establishment of neuronal networks, especially in the first postnatal weeks^13^. If embryo-derived resident microglia cells are depleted in adulthood, they repopulate from surviving endogenous cells^18^. D*e novo* establishment of microglial networks occurs by surviving cells through clonal expansion^19^ and these postnatal-derived repopulated microglial cells exhibit a different transcriptional signature^20^. However, whether adulthood-derived repopulated microglia exhibit functional differences relative to original embryo-derived resident populations is hotly disputed.

There is evidence that immune system activation, such as during illness, results in increased sleepiness, suggesting an interaction between the immune system and the sleep-wake cycle^21,22^. Brain-resident macrophages, being the closest immune components to sleep-regulating centers in the brain, might play a crucial role in this interaction. While some studies have demonstrated a clear influence of sleep and sleep loss on resident microglia^23–26^, the reverse relationship—how, and to what extent, brain-resident macrophages modulate the sleep–wake cycle and vigilance states—remains only partially understood and is still an emerging area of research. Recent data indicate that genetic and pharmacological depletion of microglia increases time spent asleep^27–32^. Liu et al., using a genetic microglia depletion model, showed that microglia-depleted mice exhibited reduced total wakefulness and increased total NREM sleep during the dark period. They attributed this to unstable wakefulness, due to the change in thalamic reticular nucleus neuronal activity and ceramide production, resulting in more frequent transitions from wakefulness to NREM sleep^32^. Further studies by other groups have confirmed these findings, reporting increased NREM sleep following microglia depletion^27–30^, while no significant changes were observed in REM sleep^28–30^. Alterations in normal sleep patterns have also been reported after microglia depletion in disease and inflammatory conditions^27,30,31^. Following microglia repopulation, it was reported that NREMS and wakefulness largely returned to normal level^28,30^. The mechanisms by which brain-resident macrophages contribute to the regulation of brain vigilance states remain largely unexplored. However, recent findings suggest that microglia influence sleep through several mechanisms, including norepinephrine transmission^33^, TNF-α signaling^34^, and synaptic^29^ or metabolite regulation^32^.

To further advance the field, it is essential to thoroughly investigate the extent to which brain-resident macrophages influence sleep states and brain activity during these states. Comprehensive EEG-recorded brain activity analysis will therefore be an important indicator of the effects of the immune system on brain oscillations. Additionally, the influence of brain macrophages on sleep-regulating neurotransmitters and synaptic connectivity warrants further exploration. Finally, it remains unclear whether adulthood-derived repopulated brain-resident macrophages are capable of modulating neural circuitries in the same manner as resident cells, including synaptic plasticity and neurochemical balance, which are crucial for normal sleep regulation.

Here, we undertook to address these questions through a sound experimental design and in-depth analyses. Using a comprehensive characterization of EEG signals recorded from the mice brain, we first explored whether the absence of resident brain-resident macrophages influences sleep-related brain oscillatory patterns. Next, we studied whether repopulated postnatal macrophages can normalize these patterns. Finally, we investigated how the absence and re-presence of microglia and CAMs affect brain network connectivity and neurochemistry. Our data show functional differences between resident and postnatally adulthood-derived repopulated brain-resident macrophages.

## Results

### Repopulated brain-resident macrophages are deficient in rebuilding normal NREMS circuitry

**Fig. 1:**
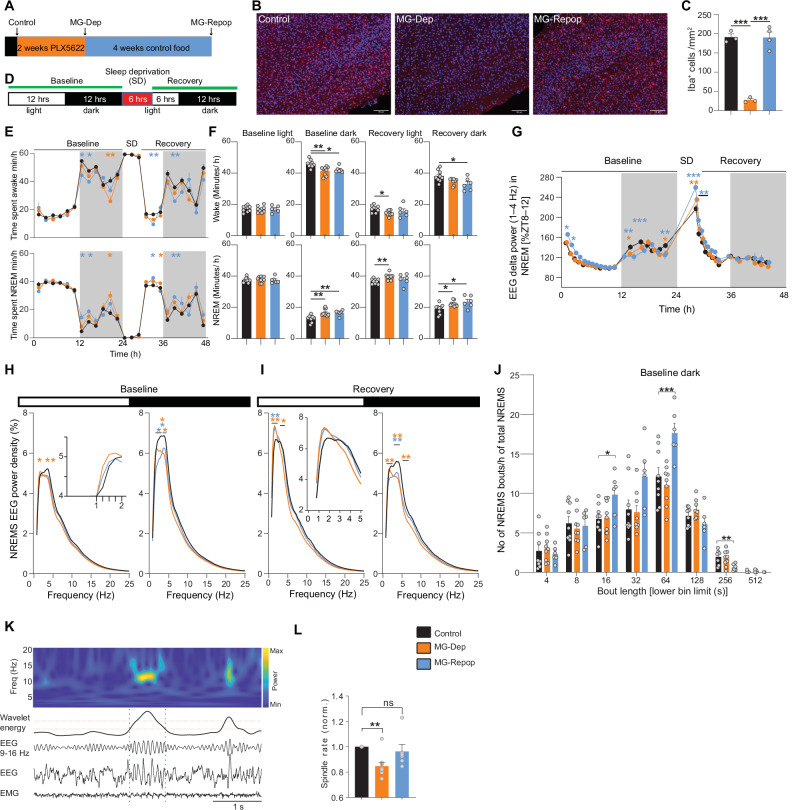
Essential role of MG in regulation of NREMS and associated EEG oscillations. **A** Schematic illustration of the experimental procedure conducted on adult mice to assess the brain’s electrical activity using EEG. Recordings prior to PLX5622 drug administration served as controls. Mice were then fed PLX5622-containing food during 2 weeks. During the final 3 days of this 2-week period, a 2nd series of recordings was conducted. Following PLX5622 treatment, mice were provided control food for 4 weeks. After 4 weeks under normal food, a 3rd recording was performed. All 3 recordings were performed on the same mice. **B**, **C** PLX5622 substantially depleted the microglia from the brain within 2 weeks (compare control and MG-Dep). Return to normal food repopulated microglia into the brain after 4 weeks (compare MG-Dep and MG-Repop) (**C**): for this analysis 3, 3, and 4 mice were used in the control, MG-Dep, and MG-Rep conditions, respectively). **D** Recording paradigm. The first 2 days of recordings served as baseline, averaged as one day. The 2^nd^ day consisted of 6 h SD followed by 18 h of recovery. **E** Time-course of vigilance states; Wakefulness (top) and NREMS (bottom). Compared to controls (black datapoints), MG-Dep (orange datapoints) and –Repop (blue datapoints) mice show a large increase in NREMS duration during the dark period both at baseline and during recovery (*n* = 9 for control and MG-Depleted conditions and *n* = 6 for MG-Repopulated condition, 2-way ANOVA, followed by Dunnett’s test, *P < 0.05; **P < 0.01; ***P < 0.001, orange and blue stars, significant differences between control and MG-Dep and MG-Repop mice, respectively). Data points are shown in minutes per hour (min/h) and represent the average of 2 h. (**F**) From left to right: Time in wakefulness (top) and NREMS (bottom) during baseline light, dark and recovery light and dark periods (Mixed-effects analysis, followed by Dunnett’s test, *P < 0.05; **P < 0.01; ***P < 0.001). (**G**) Time-course of EEG delta power (1–4 Hz) during the 3-day recording. EEG delta power is a proxy of sleep need. Both MG-Dep and MG-Repop mice show deficiency in building up sleep need during the dark period (2-way ANOVA, followed by Dunnett’s test, *P < 0.05; **P < 0.01; ***P < 0.001, orange and blue stars, significant differences between control and MG-Dep and MG-Repop mice, respectively). **H** EEG power spectra of experimental mice in NREMS during baseline light (left) and dark (right) periods. EEG power is expressed as % of baseline power of control condition. Insets show amplification of specific EEG frequencies (2-way ANOVA, followed by Dunnett’s test, *P < 0.05; **P < 0.01; ***P < 0.001, orange and blue stars indicate significant differences between control and MG-Depleted and MG-Repopulated mice, respectively). **I** EEG power spectra of experimental mice in NREMS during recovery light (left) and dark (right) periods. **J** Distribution of NREMS bout durations during baseline dark period across the experimental mice. Sleep bout duration is represented on the x-axis (1-way ANOVA, followed by Tukey test). **K** (Top) Representative time-frequency heatmap of spindles. (Bottom) Representative EEG/EMG signals of a detected spindle. Dashed horizontal lines indicate upper and lower thresholds used to detect spindles with wavelet energy within 9–16 Hz, and dashed vertical lines indicate the start and end of the detected spindle. **L** Spindle rate during NREMS episodes in different conditions. Spindle rate significantly decreased in MG-depleted mice (mixed-effects analysis, followed by Dunnett’s test, *P < 0.05; **P < 0.01; ***P < 0.001)). **D**–**L**: *n* = 9 for control and MG-Depleted conditions and *n* = 6 for MG-Repopulated condition.

We examined to what extent embryo-derived brain-resident macrophages contribute to sleep regulation and whether postnatal-derived repopulated macrophages mirror the same functionality. To address this, we depleted microglia from the brains of mice for two weeks using colony-stimulating factor 1 receptor (CSF1R) inhibitor PLX5622^35^, and then allowed them to repopulate for four weeks (Fig. 1A). CSF1 receptor is vital for microglia survival. Antagonizing it severely depletes microglia within three days, and upon the termination of treatment, repopulation can be readily observed within a few days^20^. We observed substantial microglia depletion (>86%) after two weeks of PLX5622 treatment, and complete repopulation four weeks after withdrawal (Fig. 1B, C).

We recorded EEG signals before depletion (control), after depletion and after repopulation (Fig. 1A). Since CSF1 inhibition also depletes CAMs^36^, the effects we report here are due to the depletion of both cell types, which we refer to collectively as MG. Our experimental conditions therefore included control, MG-Dep (microglia/CAM depleted), and MG-Repop (microglia/CAM repopulation). To evaluate the distribution of vigilance states, we employed our standard sleep/wake phenotyping protocol^3^, with two days of baseline (which were averaged and presented as one day in the figures) and in the third day mice underwent 6 h of SD (ZT0-ZT6) and 18 h of recovery (Fig. 1D). In baseline conditions, all experimental mice demonstrated the characteristic cycling behavior, with higher levels of wakefulness during the dark period and higher levels of NREMS during the light period and exhibited an increase in NREMS amounts in response to SD (Fig. 1E, F). However, a significant NREMS increase was observed in the baseline dark period when comparing the MG-Dep to the control condition. Interestingly, the repopulation of MG did not normalize this effect (Control: 12.75 ± 0.72 min, MG-Dep: 16.78 ± 0.77 min, MG-Repop: 16.54 ± 0.70 min, mean ± SEM) (Fig. 1E, F, bottom panels). Reciprocally, there was a notable reduction in wakefulness during the dark period (Fig. 1E, F, top panels).

To gain insight into sleep homeostatic regulation in different mouse groups and determine whether increased sleep is due to increased need for sleep, we analyzed the time course of NREMS EEG delta power (1-4 Hz, a recognized quantitative readout of sleep need) across our 3-day recordings. All mice groups showed a typical decline in delta power during the light period and a strong rebound after SD, indicating homeostatic regulation of sleep need in all groups (Fig. 1G). However, the buildup in delta power during the baseline dark period was significantly lower in MG-Dep and MG-Repop mice compared to WT mice (Fig. 1G). This attenuation was may be partly related to the increased amount of sleep during the baseline dark period (Fig. 1E, lower panel). Additionally, when mice were exposed to enforced wakefulness (SD), the EEG delta power increase was significantly more pronounced in MG-Dep and MG-Repop than in control conditions (Fig. 1G). This indicates that sleep pressure following sleep deprivation was elevated in MG-Dep and MG-Repop mice relative to controls. Immediately after sleep deprivation, mice fall asleep and the elevated delta power in both MG-Dep and MG-Rep mice began to decline. While this decline occurred rapidly in MG-Dep mice, it was slower in MG-Rep mice, taking approximately three hours (corresponding to four data points in Fig. 1G) to return to the control delta power levels (Two-way ANOVA; Interaction F (62, 651) = 3.29, P < 0.0001). Altogether, these data indicate impaired buildup of homeostatic sleep need during the waking period.

Spectral analysis of NREMS revealed that MG-Dep mice exhibited higher slow-delta (0.75 – 1.25 Hz, Two-way ANOVA, experimental condition: F (2, 4137) = 15.64, *P* < 0.0001) activity compared to control mice in baseline light period (Fig. 1H, left). This frequency band appears particularly affected also in MG-Repop mice, as it was significantly increased in the recovery light period following SD (Two-way ANOVA, experimental condition: F (2, 4137) = 18.58, *P* < 0.0001, Fig. 1I, left). The fast-delta (3.5–4 Hz) frequency range was in contrast blunted during both baseline and recovery light periods only in the MG-Dep condition (Fig. 1H, I, left). During the baseline dark period, large delta power blunting was observed in both MG-Repop (1.5-3.5 Hz) and MG-Dep (2.25–4 Hz) conditions (Two-way ANOVA, experimental condition: F (2, 4137) = 31.16, *P* < 0.0001). These deficits remained nearly the same during the recovery dark period (Two-way ANOVA, experimental condition: F (2, 4137) = 60.62, *P* < 0.0001), indicating that NREMS of both experimental mice appears to be shallower (Fig. 1H, I, right). Collectively, it can be concluded that the presence of MG is necessary for the precise regulation of fast and slow-delta power as a proxy of the sleep homeostatic process. MG repopulation not only fails to restore NREMS regulation but worsens it.

To determine whether increased NREMS duration is due to enhanced state initiation (increased number of NREMS episodes) and/or to enhanced state maintenance (increased NREMS episode duration), we conducted a sleep fragmentation analysis. We found that while MG-Dep mice have similar sleep bout duration patterns as the control condition, MG-Repop mice showed a larger number of shorter NREMS bouts (16 seconds [Control: 6.7 ± 0.63, MG-Dep: 6.8 ± 0.7, MG-Repop: 9.84 ± 1.12]) and 64 seconds [Control: 12.12 ± 1.15, MG-Dep: 11.02 ± 0.81, MG-Repop: 17.63 ± 1.2]) and fewer longer NREMS bouts (>256 seconds [Control: 1.96 ± 0.28, MG-Dep: 1.74 ± 0.33, MG-Repop: 0.62 ± 0.17]), suggesting a strong destabilization of NREMS in the MG-Repop brain (Fig. 1J).

During NREMS, brief bursts of brain activity in the 9-16 Hz frequency range, called spindles, are generated by thalamo-cortical interactions and play roles in processes such as learning, memory, and cognition^37^. To determine if MG depletion and repopulation influence spindle generation, we quantified spindle rate during NREMS episodes and found a significantly reduced spindle rate in MG-Dep mice compared to the control condition (Fig. 1K, L; see “Methods”). In contrast to other features of NREMS, MG repopulation was found to normalize the occurrence rate of spindle events, indicating that MG repopulation partially rescues the circuit deficits seen after MG depletion.

Altogether, these data suggest that embryo-derived resident MG are an integral part of NREMS regulation and maintenance, and repopulated postnatal MG largely fail to fulfill the normal NREMS regulatory functions of resident MG.

### Depletion of brain-resident macrophages prolongs REMS and degrades its quality

**Fig. 2:**
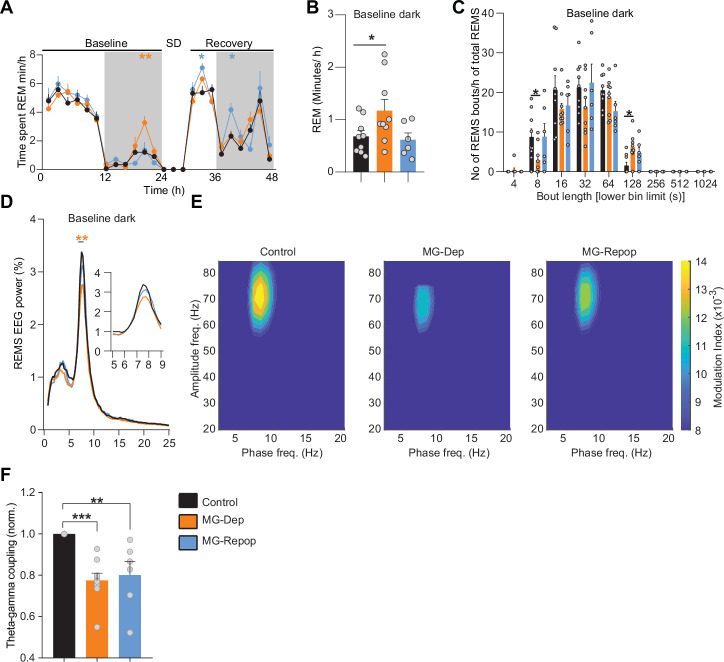
REMS regulation largely depends on the presence of the MG. **A** Time-course of REMS duration in control (black), MG-Dep (orange) and MG-Repop (blue) mouse groups. MG-Dep mice exhibit a large increase in REMS time during the baseline dark period (*n* = 9 for control and MG-Dep conditions and *n* = 6 for MG-Repop condition, 2-way ANOVA, followed by Dunnett’s test, *P < 0.05; **P < 0.01; ***P < 0.001, orange and blue stars represent significant differences between control and MG-Dep and MG-Repop mice, respectively). Data points are shown in minutes per hour (min/h) and represent the average of 2 h. **B** Time in REMS during baseline dark period (mixed-effects analysis, followed by Dunnett’s test, *P < 0.05; **P < 0.01; ***P < 0.001). **C** Distribution of REMs bout durations during baseline dark period across 3 mouse groups. Sleep bout duration is presented as x-axis (1-way ANOVA, followed by Tukey test). **D** EEG power spectra of all experimental mice in REMS during baseline dark period. Insets show amplification for specific EEG frequencies (2-way ANOVA, followed by Dunnett’s test, *P < 0.05; **P < 0.01; ***P < 0.001, orange and blue stars are significant differences between control mice and MG-Dep and MG-Repop mice respectively). **E** Comodulogram graphs show the modulation index for a wide range of frequency pairs, obtained from 12-h recordings of one animal in different conditions. **F** Normalized theta-gamma coupling across 3 experimental conditions. Theta-gamma coupling is significantly decreased in MG-depleted and -repopulated conditions (mixed-effects analysis, followed by Dunnett’s test, *P < 0.05; **P < 0.01; ***P < 0.001)). **A**–**F**: *n* = 9 for control and MG-Depleted conditions and *n* = 6 for MG-Repopulated condition.

Different neural pathways and neurotransmitters regulate REMS compared to NREMS. However, the role of MG in the regulation of REMS is not well understood. We quantified REMS amount across the dark/light periods and found that MG-Dep mice display more REMS compared to the control and MG-Repop conditions during the baseline dark period (Control: 0.67 ± 0.1, MG-Dep: 1.17 ± 0.21 min/h, mean ± SEM). However, in contrast to NREMS, MG repopulation corrected the abnormal expression of REMS (MG-Repop: 0.61 ± 0.13 min/h) (Fig. 2A, B). To investigate if the increase in REMS is due to the consolidation of this state, we performed fragmentation analysis and found that the number of shorter REMS bouts (8 s) is largely decreased (Control: 8.7 ± 2.05, MG-Dep: 2.8 ± 1.2, MG-Repop: 8.8 ± 3.3) and longer bouts (>2 min) are increased (Control: 1.5 ± 0.8, MG-Dep: 6.1 ± 1.1, MG-Repop: 4.71 ± 1.4, mean ± SEM) (Fig. 2C) in MG-Dep mice, suggesting stabilization of REMS in the absence of MG.

Theta oscillations dominate the EEG signal during REMS, where theta phase dynamically modulates gamma amplitude in hippocampal and cortical networks^38^. We next investigated the contribution of MG in theta oscillations and theta-gamma interactions. Spectral analysis of REMS revealed a significant dampening of theta (6–8 Hz) power in MG-Dep mice during the baseline dark period (Fig. 2D), which was restored after MG repopulation. We also found that the theta-gamma coupling is significantly reduced in the MG-Dep condition compared to the control. Although MG repopulation attempts to bring it back to the normal level, it fails significantly (Fig. 2E, F).

Altogether, these findings suggest that the presence of MG is necessary for the normal expression of REMS.

### Brain-resident macrophages contribute to the maintenance of active wakefulness

**Fig. 3:**
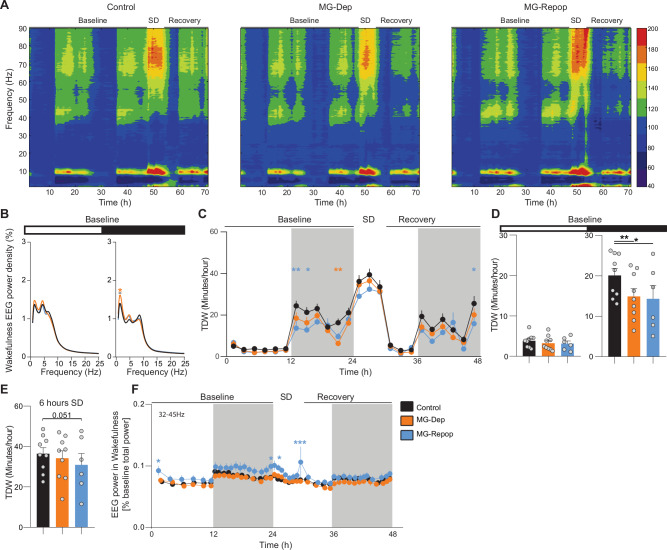
MG Repopulation fails to normalize the waking state deficits induced by MG depletion. **A** Time x frequency x power heatmap representations of the waking EEG of control, MG-Dep and MG-Repop mice across the 3 recorded days (48 h baseline, 6 h sleep deprivation and 18 h recovery) show large changes in higher frequency bands. **B** EEG power spectra of experimental mice in wakefulness during baseline light and dark periods (2-way ANOVA, followed by Dunnett’s test, *P < 0.05; **P < 0.01; ***P < 0.001, orange star are significant differences between control mice and MG-Dep mice). **C** Time-course of TDW (theta-dominated wakefulness) amounts indicates decreased TDW time during the dark period in MG-Dep and MG-Rep. Datapoints are shown in min/h and represent the average of 2 h (baseline days 1 and 2 are averaged), (2-way ANOVA followed by Dunnett’s test, orange and blue stars are significant differences between control and MG-Dep and MG-Repop mice, respectively). **D** Quantification of time in TDW (min/h) during baseline light and dark period (mixed-effects analysis, followed by Dunnett’s test, *P < 0.05; **P < 0.01; ***P < 0.001). **E** Time in TDW during SD. **F** Waking EEG slow-gamma (32–45 Hz) power dynamics during the 3-day recording (baseline days 1 and 2 are averaged) (2-way ANOVA, followed by Dunnett’s test). **A**–**F**: *n* = 9 for control and MG-Depleted conditions and *n* = 6 for MG-Repopulated condition.

The quality of prior wakefulness contributes to the expression of both NREMS and REMS. Along these lines, a specific sub-state of prior wakefulness was recently proposed to drive NREMS need^39^, and some aspects of wakefulness may functionally substitute for REMS^40^. To investigate how wakefulness is expressed in our experimental conditions, we performed a dynamic analysis of the full waking EEG spectrum across three recording days. This global time-frequency analysis revealed differential expression of the waking frequency bands during light-dark cycles across three days, and most notably during enforced wakefulness (SD) (Fig. 3A). To better quantify those changes, we analyzed the waking EEG power dynamics within specific frequency ranges of interest. Our analysis revealed that during enforced wakefulness MG-Repop mice exhibit an increase in lower delta (1–4 Hz, interaction: Two-way ANOVA, F (88, 924) = 1.328, *P* = 0.0280), slow gamma (32–45, interaction: Two-way ANOVA, F (82, 861) = 2.149, *P* < 0.0001) and (45-55, interaction: Two-way ANOVA, F (82, 861) = 2.692, *P* < 0.0001), and fast gamma (70–90 Hz, interaction: Two-way ANOVA, F (82, 861) = 1.604, *P* < 0.0009) frequency bands compared to the control mice (supplementary fig. 1, Fig. 3F). While time course analysis of specific bands did not reveal differences between control and MG-Dep mice, our PSD analysis revealed that MG-Dep mice exhibit a large increase in slow delta (1.35–1.75 Hz) activity compared to the control mice (Fig. 3B).

Mice spend a portion of their waking time engaged in exploratory and motivated behaviors, such as running, nest building, or drinking, often referred to in rodents as “active wakefulness”. These behaviors are associated with the EEG signature called “theta-dominated wakefulness” (TDW 6.0-9.5 Hz), which has been suggested to be the principal driver of the sleep homeostat^39^. We quantified TDW in our mice and found a large decrease in its amount in both MG-Dep and MG-Repop mice during the baseline dark period (Fig. 3C, D). This reduction was more pronounced in MG-Repop mice, which was also seen during SD (Fig. 3C, E). TDW is associated with heightened gamma activity. We analyzed the time course of gamma activity during wakefulness and found an increase in slow (32–45 Hz) frequency bands in the MG-Repop condition compared to the controls (Fig. 3F). The increase in these frequency bands in MG-Repop mice is also apparent on time-frequency heat maps in Fig. 3A.

Overall, these data suggest a significant imbalance in the theta/gamma-rich waking state in both the MG-Dep and MG-Repop conditions, and appearance of heightened gamma activity after MG repopulation.

### Disrupted balance of synaptic signaling upon MG depletion and repopulation

Sleep is associated with changes in synaptic plasticity^41^ and microglia play a substantial role in synaptic functions, which are dependent on neuronal activity and vigilance states (VS)^42^. To investigate whether MG depletion and repopulation induce changes in brain network connectivity at the synaptic level, we analyzed a subset of excitatory and inhibitory synaptic density proteins that are involved in sleep regulation, (e.g., the core brain molecular correlate of sleep loss, HOMER1) in the brains of our experimental mice.

**Fig. 4:**
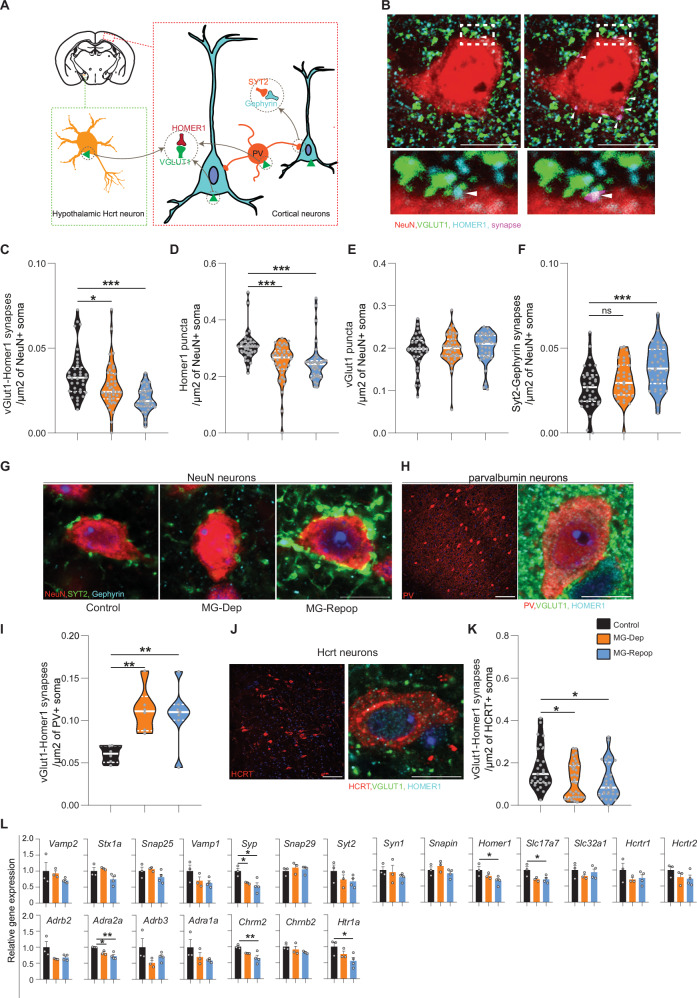
Large synaptic imbalance after MG depletion and repopulation. **A** Schematic illustration of the cell types for which the number of synapses was counted. NEUN (blue) and PV (orange) positive neurons in the cortex and HCRT neurons in the hypothalamus. The number of co-localized excitatory VGLUT1-HOMER1 synaptic proteins on NEUN, PV and HCRT positive neurons were counted. Additionally, the number of co-localized inhibitory SYT2-Gephyrin synaptic proteins on NEUN neurons were counted. **B** Representative image depicting pre, postsynaptic proteins and their colocalization in a NEUN positive neuron. Synapses (arrowheads) are marked with pink color using ImageJ macro (right). Scale bar: 10 µm. **C** Number of VGLUT1-HOMER1 synapses/μm^2^ of NEUN^+^ soma. *N* = 342 cells from 12 mice for control, 324 cells from 11 mice for MG-Dep, and 357 cells from 12 mice for MG-Repop conditions. The data show a reduced number of these synapses in both MG-Dep and MG-Repop conditions (**D**) Number of HOMER1 puncta/μm^2^ of NEUN^+^ soma. **E** Number of VGLUT1 puncta/μm^2^ of NEUN+ soma. **F** Number of SYT2-Gephyrin synapses/μm^2^ of NEUN^+^ soma. *N* = 299 cells from 12 mice for control, 270 cells from 11 mice for MG-Dep, and 306 cells from 12 mice for MG-Repop conditions. **G** Representative images depicting SYT2-Gephyrin synapses in NEUN^+^ cortical neuron. Large increase in these inhibitory synapses is observed in the MG-Repop condition. Scale bar: 10 µm. **H** Distribution of PV^+^ neurons (red) in the cortex. Scale bar: 100 µm (left), 10 µm (right). **I** Number of vGLUT1-HOMER1 synapses/μm^2^ of PV^+^ soma. *N* = 29 cells from 5 mice for control, 29 cells from 5 mice for MG-Dep, and 23 cells from 4 mice for MG-Repop conditions. **J** Left, distribution of HCRT^+^ neurons (red) in the lateral hypothalamus. Right, a HCRT^+^ neuron stained with VGLUT1 (green) and HOMER1 (Cyan). Scale bar: 100 and 10 µm. **K** Number of VGLUT1-HOMER1 synapses/μm^2^ of HCRT+ soma. *N* = 70 cells from 3 mice for control, 73 cells from 3 mice for MG-Dep, and 77 cells from 4 mice for MG-Repop conditions. **L** Gene expression of synaptic proteins and neurotransmitter receptors at RNA level following MG depletion and repopulation. *N* = 3 for control and MG-Dep and *n* = 4 for MG-Repop conditions. 1-way ANOVA, followed by Tukey test, *P < 0.05; **P < 0.01; ***P < 0.001.

Using an ImageJ macro, we counted the number of synapses on cortical and hypothalamic neurons (Fig. 4A, B) and found that the number of excitatory VGLUT1-HOMER1 synapses on NEUN^+^ cortical neurons was significantly reduced in both MG-Dep and MG-Repop conditions. This reduction was more noticeable in MG-Repop mice (Fig. 4C). Interestingly, this effect was regulated at the postsynaptic level (HOMER1-associated, Fig. 4D) rather than the presynaptic level (VGLUT1-associated, Fig. 4E). Recently, cortical PV neurons have been proposed to be responsible for homeostatic sleep regulation^43^. We found that the number of parvalbumin (PV)-originated^44^ Synaptotagmin-2-Gephyrin (Syt2-Geph) inhibitory synapses on the cortical NEUN^+^ neurons was significantly increased under the MG-Repop condition (Fig. 4F, G). This increase could possibly be due to an increase in the activity of PV neurons, as we found that the number of VGLUT1-HOMER1 excitatory synapses on PV neurons was significantly increased under both MG-Dep and MG-Repop conditions (Fig. 4H, I). We additionally investigated the changes in the count of VGLUT1-HOMER1 synapses on representative hypothalamic neurons (HCRT) and found a significant decrease in the number of VGLUT1-HOMER1 synapses on HCRT neurons in both MG-Dep and MG-Repop conditions (Fig. 4J, K).

To further investigate changes in network connectivity in the brains of MG-Dep and MG-Repop mice, we analyzed additional synaptic markers and sleep-specific neurotransmitter receptors (e.g., receptors of monoaminergic systems and neuropeptides) on RNA extracted from cortical samples of these mice. Our bulk analysis revealed that some general synaptic markers, such as *Synaptophysin I* (*Syp*), are largely downregulated in both experimental mice, while *Slc17a7* (encoding Vesicular glutamate transporter 1) and *Homer1* genes are significantly downregulated in the cortex of the MG-Repop condition. Among the specific neurotransmitter markers analyzed, the adrenergic receptor family (e.g. *Adrb2*, *Adra2a*, *Adrb3*, and *Adra1a*) genes showed a tendency to be less expressed in both MG-Dep and MG-Repop conditions, with a marked decrease in the expression of the *Adra2a* gene. Additionally, we observed a reduction in expression of *Cholinergic receptor muscarinic 2* (*Chrm2*) and the serotonergic receptor *5-Hydroxytryptamine receptor 1 A* (*Htr1a*) genes, which mainly occur in the brain of MG-Repop mice (Fig. 4L).

Taken together, these data suggest that brain connectivity at the synaptic level is largely impaired following the depletion of brain-resident macrophages. Repopulating the brain with these macrophages not only failed to normalize connectivity but, in many cases analyzed, worsened it.

### Neurotransmitter release is largely restored after the repopulation of resident macrophages

**Fig. 5:**
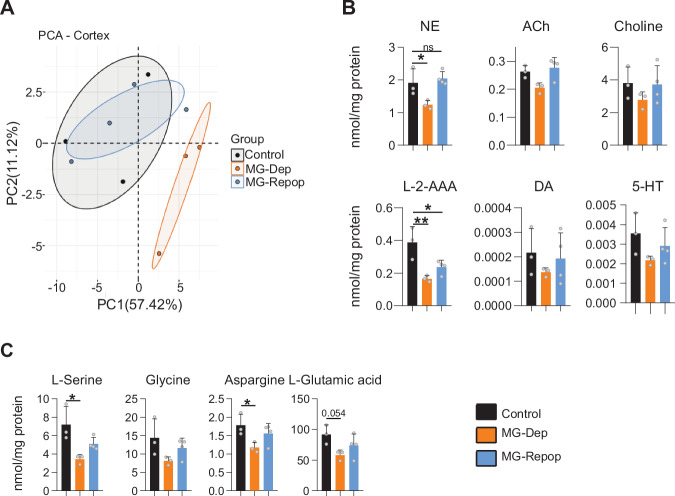
MG repopulation largely restores the metabolite changes induced by microglial depletion. **A** PCA of targeted metabolites in cortex isolated from control (*n* = 3), MG depleted (*n* = 3) and repopulated (*n* = 4) mice. **B** LC-MS/MS assessment of the neurotransmitter content in the brain of control, MG-Dep and MG-Repop mice. **C** Downregulation of amino acids in the brain of experimental mice after MG depletion. 1-way ANOVA, followed by Tukey test, *P < 0.05; **P < 0.01; ***P < 0.001.

Synapses, in addition to providing structural connections in the brain, play a crucial role in transmitting neurotransmitters essential for the function of complex neural systems. We therefore questioned whether the levels of essential neurotransmitters involved in sleep regulation are affected by the depletion and repopulation of brain-resident macrophages. We conducted targeted metabolomics to analyze the neurotransmitter content and metabolite reservoir of the cortex, target of most neurotransmitters and region where EEG signals are acquired. Assessment of neurotransmitter content was performed using chromatography with tandem mass spectrometry (LC-MS/MS) in the brain of control, as well as MG-Dep and MG-Repop mice. We found that pharmacological depletion of MG led to a distinct clustering of metabolites, as indicated by principal component analysis (PCA) (Fig. 5A). This suggests that MG plays an important role in maintaining the overall metabolic composition of the cortex. MG repopulation however restored the cortical submetabolome to a pattern similar to the one observed under control experimental conditions (Fig. 5A). Univariate analysis revealed a reduction in norepinephrine upon MG depletion, which was restored after repopulation (Fig. 5B). The concentrations of acetylcholine, choline, dopamine, and serotonin remained unchanged between the experimental conditions, with a slight tendency to decrease in MG-Dep condition.

Furthermore, we observed that MG depletion led to a marked reduction of L-2-amino adipic acid (L-2-AAA), which was only partially recovered after repopulation (Fig. 5B). We also analyzed the precursors of neurotransmitters and found a significant decrease in the amino acids L-serine and asparagine under depleted condition, which could be restored upon MG repopulation (Fig. 5C). Similarly, there was a trend towards decreased glycine and L-glutamic acid content (Fig. 5C).

Taken together, our data suggest that while network connectivity is significantly impaired in MG-Dep condition and resistant to MG repopulation, the underlying substrates (neurotransmitters and metabolites) are largely restored after MG repopulation.

## Discussion

In this study, we investigated the extent to which embryo-derived brain-resident macrophages contribute to sleep regulation and whether postnatally derived repopulated ones serve a similar role. We demonstrate that MG depletion has a significant impact on the homeostatic regulation of sleep. The brain circuits most vulnerable to microglia depletion and repopulation, causing the changes in sleep EEG that we document, are still unknown. However, changes in slow-delta activity in NREMS, state stabilization and reduced theta power in REMS, as well as changes in waking slow-delta activity, were reported in mice with defects in hypothalamic HCRT and brainstem serotonergic and noradrenergic systems^3,39,45,46^. This is suggestive of functionally important local interactions between microglial populations and sleep regulating circuits, as mentioned above.

We showed that the changes in sleep patterns after MG depletion are not fully reversed after MG repopulation. Previous studies have shown that the brain global transcriptomes are little influenced by repopulated microglia^20^. Furthermore, it has been suggested that after MG repopulation, neuroprotection is achieved through IL-6 induction by neurons^47^. Other studies reported full functionality of retinal microglia after repopulation, including continuous retinal surveillance, maintenance of synaptic structure, and normal behavioral and physiological responses to retinal injury^48^. Additionally, NREMS and wakefulness were reported to largely return to normal level following microglia repopulation^28,30^. However, our data reveal that MG repopulation not only fails to completely restore various aspects of sleep regulation, but in some cases exacerbates altered parameters, such as fragmentation of NREMS and dysregulation of sleep need. This demonstrates functional differences between resident and repopulated microglial cells. We speculate that not all microglia-neuronal interactions are properly reestablished by adulthood-derived repopulated microglia after repopulation. Whether a longer repopulation period might correct the abnormalities observed in our analysis warrants further investigation. We observed a significant imbalance between excitatory and inhibitory synapses after depletion of resident microglia and after repopulation with adult microglia, with a notable increase in inhibitory synapses following repopulation. Studies in juvenile mice previously reported that microglia depletion in the developing brain significantly increases the number of both excitatory (VGLUT1-HOMER1) and inhibitory (Syt2-Gephyrin) synapses on cortical neurons, an effect that persisted after repopulation until P30. However, in the adult (P60) brain, synapse density returned to normal control levels, suggesting that depleting microglia during development causes long-lasting, albeit not permanent, effects^49^. Our data, however, show that depleting microglia in adulthood, and then repopulating them, induces different results, with a reduction in excitatory synapses in both MG-Dep (reported also before^29^) and MG-Repop conditions, and an increase in inhibitory synapses in MG-Repop conditions. In the developing brain, it has been suggested that MG-Dep impairs synaptic pruning processes and alters related gene expression^13,49^. Whether the same process or any specific pathways are induced by MG depletion and repopulation in the adult brain, such as aberrant reactivation of developmental programs^50^, remains to be found.

Furthermore, other studies have suggested that microglia play a role in restraining excessive neuronal activation that cannot be sufficiently suppressed by inhibitory neurons alone^51^. Our results suggest that embryo-derived resident microglia also control excessive activity of inhibitory PV neurons, but the reasons why this is not corrected with adulthood-derived repopulated microglia needs further investigation.

Deficient synaptic neurotransmission was proposed as a potential cause for abnormal sleep regulation^52^. We found here an imbalanced neurotransmitter and metabolite content of the brain in mice devoid of prenatal microglia. It appears that norepinephrine neurotransmission is particularly sensitive to MG deficiency, as both the levels of norepinephrine metabolites and the corresponding receptors are significantly affected by MG depletion. Norepinephrine is implicated in both wakefulness^53^ and sleep regulation^33,54,55^, and it has been suggested that microglial surveillance process relies heavily on norepinephrine signaling^56,57^. Additionally, we observed a decrease in the expression of neurotransmitter precursors such as L-serine, which was mirrored by changes in the concentrations of glycine, asparagine, and glutamic acid. Previous studies have proposed serine as a link between metabolism and neurotransmission^58^, and serine serves as a precursor for synthesizing ceramide^59^, which has been suggested to be essential for maintaining stable wakefulness^32^.

Our findings indicate that MG affect vigilance state regulation via multiple pathways and play a more substantial role in maintaining the physical connectivity of brain networks rather than neurochemical connectivity, as MG repopulation largely restores metabolites but not synapses. To gain a detailed understanding of MG regulation of sleep, it will be necessary to explore local specific microglial depletion, and disruption of microglia-neuron interactions using inducible systems. Additionally, investigating the separate contributions of microglia and CAMs in sleep regulation using Cre lines under the promoter of *Hexb*^60^ and *Mrc1*^61^ genes, respectively, would be a starting point. The observed vigilance state phenotypic alterations may also be influenced by disruptions in central factors (such as protein phosphorylation orchestrated by microglial TNF-α)^34^, and peripheral players (since CSF1R inhibition also effects circulating and tissue macrophages)^62^ warranting further investigations.

Potential limitations of our study include the possible off-target effects of PLX5622. While tissue macrophages and circulating monocytes express CSF1R, their survival depends not only on CSF1/CSF1R signalling but also on CCL2/CCR2 signalling—pathways that are not present in microglia^63^. Therefore, it is generally believed that systemic delivery of CSF1R inhibitors predominantly targets CNS microglia and does not significantly affect the development or function of peripheral macrophages^64–66^. However, some uncertainty still remains. To definitively rule out the influence of potential off-target effects on the observed sleep phenotype, future studies employing genetic approaches to selectively deplete brain macrophages without affecting peripheral tissues are warranted. Another limitation of our study is the exclusive use of male animals, which restricts the generalizability of our findings to females. Future studies involving both sexes are necessary to determine whether the role of brain-resident macrophages in sleep regulation is consistent across genders.

Overall, our results shed new light on the complex interaction between the neuroimmune system and sleep regulation, and they pave the way for future investigations in this important physiopathological field.

## Methods

### Animals

Wild type (C57BL/6 J) male mice were used in this study. All animals were between 12 and 14 weeks old during the time of experiments and animal experiments were approved by the Ministry for Nature, Environment and Consumers‘ Protection of the state of Baden-Württemberg and were performed in accordance to the respective national, federal, and institutional regulations. At all times, care was taken to minimize animal discomfort and avoid pain. Animals were housed under standard conditions in appropriate cages with suitable rodent litter, and had ad libitum access to food and water. They were maintained on a 12:12 h light-dark cycle (lights on at 08:00) in an experimental room with controlled temperature (25 °C), humidity (50–60%), and light intensity (70–90 lux).

### Gene expression analysis

For qPCR analysis, RNA extracted from the cortical samples of control, MG-Dep and MG-Repop mice. RNA was extracted using RNeasy micro kit (74004) and cDNA synthesis was performed using random hexamer primers and M-MLV Reverse Transcriptase Promega kit (M3682). SYBR green-based and Taqman probes were used to detect the gene expression. The assay was performed using a LightCycler480 (Roche).

### Immunofluorescence and synapse counting

Mice were anesthetized with ketamine (100 mg per kg body weight) and xylazine (10 mg per kg body weight) followed by perfusion with 1× PBS. For histology, brains were kept overnight in 4% paraformaldehyde (PFA). Brains were then dehydrated in 30% sucrose for about 48 hours and then snap-frozen on dry ice. Cryosections (14μm for synapse counting and 20μm for microglia and CAM staining) from brain tissue were prepared as free floating in PBS. The following antibodies were used to stain microglia and synapses: Rabbit-anti-IBA1 (ab178846, abcam), anti-NEUN (ab177487, ab104224, abcam), anti-VLUT1 (135304, synaptic system), anti-HOMER1 (ABN37, Millipore), anti-Gephyrin (147318, synaptic system), anti-Synaptotagmin-2 (ZDB-ATB-081002-25, ZFIN), anti-Parvalbumin (MAB1572, Merck) and anti-HCRT-A (SC-8070). The DAPI was used to stain the nucleus. Iba1 antibody was incubated overnight at 4 °C and the primary antibodies to detect synapses were incubated for 1.45 h at room temperature. The brain cuts were incubated with secondary antibodies for 1–2 h at room temperature. Fluorescence imaging was performed with TCS SP8 X (Leica Microsystems). Imaging of synapses was performed using 63X objective with zoom factor 8. Iba1^+^ cells were counted using Fiji. Synapse analysis and quantification were conducted using Fiji macro developed previously^49^ with little modifications.

### Quantitative profiling of metabolites by liquid chromatography and mass spectrometry (LC-MS/MS)

Mice brain tissue biopsies were thawed and homogenised with ice-cold PBS supplemented with 1% protease inhibitor cocktail (Sigma Nr. P8340-5ML), using a cordless pestle motor and disposable pellet mixers (VWR Nr. 47747-366). Buffer volume was adjusted to reach a target of 0.1–0.3 mg wet-tissue/mL lysis buffer. Whole tissue homogenates were aliquoted for downstream measurement of total protein concentration, flash-frozen and stored at −80 ^o^C. Sulfur-containing metabolites as well as creatinine, S-adenosylmethionine and S-adenosylhomocysteine were determined according to a previously published procedure^67,68^. Lactate, TCA and glycolysis intermediates and other organic acids, and folates were determined as described in previous work^69^. Amino acids and neurotransmitters were determined using a previously described protocol^70,71^. A commercially available standardized amino acid mixture was utilized to generate a calibration curve for amino acids (Amino acid standards, physiological, Sigma, Nr. A9906-10ML). Calibration curves for all other metabolites were prepared from individual stock solutions prepared in house. Quantitation accuracy was examined by monitoring homocysteine and methylmalonic acid concentrations in an external quality control, namely, the Control Special Assays in Serum, European Research Network for the evaluation and improvement of screening, diagnosis, and treatment of Inherited disorders of Metabolism (ERNDIM) IQCS, SAS-02.1 and SAS-02.2 from MCA Laboratories, Winterswijk, Netherlands. For all other metabolites, quantitation trueness was tested by examining metabolite concentrations in plasma from a previously validated sample isolated from a healthy control individual with respect to standard reference ranges, using the same calibration curves and LC-MS/MS running conditions. Quantification of metabolites was carried out with Analyst® 1.7.2 software, 2022 AB Sciex. Principal Component Analysis (PCA) was performed using the prcomp() function in R, with data centered and scaled to account for differences in metabolite concentration ranges. Group separation and clustering were visualized using fviz_pca_ind() and fviz_pca_biplot() functions from the factoextra package, with 95% confidence ellipses added to assess variance between experimental groups. The contributions of individual metabolites to each principal component were quantified using get_pca_var(), allowing for identification of key variables driving group differences.

### Treatments

In order to eliminate cells dependent on CSF1R (MG) mice were administered a CSF1R inhibitor called PLX5622 (Plexxikon). The inhibitor was combined with AIN-76A standard chow at a concentration of 1,200ppm (Research Diets) and the mice were given unrestricted access to it for two weeks. The control group received AIN-76A standard chow without any modifications. After two weeks the PLX5622 food was replaced with control food to start repopulation of MG.

### Mice surgery, data acquisition and vigilance state analysis

EEG electrodes were implanted through the skull, and EMG electrodes were placed in the nuchal muscles of mice^72^. Surgeries were conducted at 9–12 weeks of age under deep anaesthesia (ketamine/xylazine intraperitoneally, 75 and 10 mg/kg). Following surgery, animals were given 5–7 days to recover before cables were attached to the head-mounted connectors, linking them to an EMBLA™ recording system. After an additional 5–7 days for cable habituation, data acquisition was carried out in each animal’s individual home cage. Data were analyzed using Somnologica-3™ software (Medcare). MATLAB scripts were created to quantify various parameters related to wakefulness, NREMS and REMS. These parameters include time distribution and amount of episodes, as well as the fragmentation of vigilance states, all of which were defined based on previously established criteria^3^. TDW analysis was performed using an algorithm developed by Vassalli and Franken^39^, which analyzes the EEG of each 4-second epoch scored as wake and identifies those that meet the TDW-defining criteria. If the frequency bin with the highest power density in the 3.5–15 Hz range is within the 6.5–12.0 Hz range, and the ratio of θ power in the TDW θ peak frequency ±1 Hz range to the total power across 3.5–45 Hz is greater than 0.228, then the epoch is scored as TDW, provided the following three criteria are met: (i) the epoch immediately preceding was scored as “W” or “TDW”; (ii) the epoch immediately following was not scored as SWS; and (iii) single TDW epochs that are preceded and followed by three W epochs are excluded. The 0.228 θ-to-total power ratio was chosen because TDW epochs identified algorithmically using this threshold yielded the best match with epochs visually identified as active waking based on their stereotypical, regular EEG θ rhythmic signal, in mice.

### Sleep recording

Sleep was recorded over three consecutive 24 h light-dark (LD) cycles. The first 48 h were recorded as the baseline condition. On day 3, mice were sleep-deprived from Zeitgeber Time 0 (ZT0) to ZT6, followed by an 18-hour recovery period. Recordings were conducted at three time points: (1) Control—3 days prior to drug treatment; (2) Post-microglia depletion—during the final three days of the depletion phase; and (3) Post-repopulation—during the final three days of the repopulation phase.

### Sleep deprivation

Male mice were individually housed and subjected to a 6-hour period of sleep deprivation (SD), typically initiated at light onset (ZT0, 8:00 AM) to coincide with the beginning of their rest phase, when sleep pressure is naturally high. Sleep deprivation was achieved using the gentle handling method. During this procedure, mice remained undisturbed in their home cages, connected to recording cables, and were continuously monitored for behavioral signs of sleep. Interventions to prevent sleep were introduced only when necessary and included placing paper tissue into the cage, changing the litter, gently tapping the cage, or bringing a pipette close to the animal. After 3 h, the cages were also changed. These stimuli were chosen to minimize stress and novelty, particularly toward the end of the SD period, as such factors can interfere with recovery sleep. Cage changes, individual housing, and even gentle handling can still induce stress; however, we took measures to minimize these factors as much as possible, and the treatment was applied equally to both control and experimental groups. Therefore, we consider the potential impact of these variables on the interpretation of our findings to be minimal.

### Power spectral analysis

Using Somnologica-3TM (Medcare) software, each 4 s epoch of the EEG signal was subjected to discrete Fourier transform to determine EEG power density (spectra 0–90 Hz at a frequency resolution of 0.25 Hz). We used artifact-free, same-state–flanked 4 s epochs to calculate the mean EEG spectral profiles for each behavioral states and time intervals. To account for differences among animals in absolute EEG power, the mean values of spectral profiles were converted into percentages relative to a baseline EEG power reference value of 100%. This reference value was determined for each mouse by summing the power within the frequency range of 0.75–47.5 Hz across all states and throughout the first day of baseline recordings. To control for differences in the amount of wakefulness, NREMS and REMS, this reference was weighted so that the relative contribution of each state was identical for all mice^72^. Time-frequency heatmaps of EEG power during wakefulness were generated using methods described previously^73^.

### Time-course analysis

To examine the dynamic changes of EEG power within specific frequency ranges throughout the day and night, the time-course of the activity of that frequency band was computed for 4 s epochs scored as state of interest (NREMS, REMS and wake). For this purpose, the epochs for each state were divided into percentiles, ensuring that each percentile contained a roughly equal number of epochs. The number of percentiles for NREMS and REMS were: 12 for light periods, 6 for dark periods, and 8 for the 6 h light period after SD; for waking state: 6 for light periods, 12 for dark periods, 8 for the 6 h light SD, and 4 for the 6 h light after SD. The average EEG power within the specified frequency range was computed for each percentile, and subsequently normalized based on the type of analysis. This normalization was either done relative to the total power of the baseline state or with respect to the power achieved within the delta frequency band during slow-wave sleep at ZT8-12. Like power density analysis, single epochs were excluded and only power values of the epochs that themselves, as well as the two adjacent ones, were scored as artifact-free same-state were included in the analysis.

### Theta-gamma cross-frequency coupling

We used the modulation index (MI) to measure the theta-gamma phase-amplitude coupling^38,74^. Using finite impulse response filters with an order equal to three cycles of the low cutoff frequency, we first bandpass-filtered EEG signals into theta (6–11 Hz) and fast-gamma (54–90 Hz) in both forward and reverse directions to eliminate phase distortion. We then estimated instantaneous phase of theta and the envelope of fast-gamma using the Hilbert transform. Theta phase was discretized into 18 equal bins (*N* = 18, each 20°) and the average value of fast-gamma envelope within each bin was calculated. The resulting phase-amplitude histogram (*P*) was compared with a uniform distribution (*U*) using the Kullback-Leibler distance, ${D}_{{KL}}\left(P,U\right)=\mathop{\sum }_{j=1}^{N}P\left(j\right)* \log \left[P\left(j\right)/U\left(j\right)\right]$, and normalized by *log(N)* to obtain the modulation index, *MI* = *D*_*KL*_ */ log(N)*. To explore possible coupling patterns between different pairs of low and high frequency bands, we used the comodulogram analysis^74^. We considered 16 frequency bands for phase (1–18 Hz, 1 Hz increments, 2 Hz bandwidth), and 14 frequency bands for amplitude (15–90 Hz, 5 Hz increments, 10 Hz bandwidth). MI values were then calculated for all these pairs to obtain the comodulogram graph.

### Detection of sleep spindles

NREMS spindles were detected automatically using an optimized wavelet-based method as previously described^37^. Briefly, the power of EEG signals within 9–16 Hz was estimated using the complex B-spline wavelet function, and smoothed using a 200 ms Hanning window, and then a threshold equal to 3 STDEV (STDEV: standard deviation) above the mean was applied to detect the potential spindle events. Events shorter than 400 ms or longer than 2 s were discarded. Using band pass-filtered EEG signals in the spindle range (9–16 Hz), we automatically counted the number of cycles of each detected event and excluded those with <5 cycles or more than 30 cycles. To discard artefacts, events with a power in the spindle band lower than 6–8.5 Hz or 16.5–20 Hz power bands were not included.

### Statistical analysis

The specific number (n) of replicates employed in each experiment is provided in the corresponding figure legends. The individuals responsible for conducting SD and scoring the sleep recordings were unaware of the experimental condition of the animals, ensuring blinding during these procedures. The results are presented as mean ± SEM or mean ± STDEV. Statistical analyses were conducted using GraphPad Prism. The significance of comparisons was determined using one- or two-way ANOVA or mix effect model followed by relevant post hoc tests and are reported in the figure legends.

### Reporting summary

Further information on research design is available in the Nature Portfolio Reporting Summary linked to this article.

## Supplementary information

Supplementary Information

Description of Additional Supplementary Materials

Supplementary Data 1

Reporting summary

## Data Availability

All data are available in Supplementary Data 1.

## References

[1] Ramar, K. et al. Sleep is essential to health: an American Academy of Sleep Medicine position statement. *J. Clin. Sleep. Med.*10.5664/jcsm.9476 (2021). 34170250 10.5664/jcsm.9476 PMC8494094

[2] Borbély, A. A. A two process model of sleep regulation. *Hum. Neurobiol.* **1**, 195–204 (1982). 7185792

[3] Seifinejad, A., Li, S., Possovre, M. L., Vassalli, A. & Tafti, M. Hypocretinergic interactions with the serotonergic system regulate REM sleep and cataplexy. *Nat. Commun.* **11**, 6034 (2020). 33247179 10.1038/s41467-020-19862-y PMC7699625

[4] Xu, M. et al. Basal forebrain circuit for sleep-wake control. *Nat. Neurosci.* **18**, 1641–1647 (2015). 26457552 10.1038/nn.4143 PMC5776144

[5] Osorio-Forero, A. et al. Infraslow noradrenergic locus coeruleus activity fluctuations are gatekeepers of the NREM-REM sleep cycle. *Nat. Neurosci.* **28**, 84–96 (2025). 39587312 10.1038/s41593-024-01822-0

[6] Gilestro, G. F., Tononi, G. & Cirelli, C. Widespread changes in synaptic markers as a function of sleep and wakefulness in Drosophila. *Science* **324**, 109–112 (2009). 19342593 10.1126/science.1166673 PMC2715914

[7] Maret, S. et al. Homer1a is a core brain molecular correlate of sleep loss. *Proc. Natl. Acad. Sci. USA* **104**, 20090–20095 (2007). 18077435 10.1073/pnas.0710131104 PMC2148427

[8] Saper, C. B., Chou, T. C. & Scammell, T. E. The sleep switch: hypothalamic control of sleep and wakefulness. *Trends Neurosci.* **24**, 726–731 (2001). 11718878 10.1016/s0166-2236(00)02002-6

[9] Amann, L., Masuda, T. & Prinz, M. Mechanisms of myeloid cell entry to the healthy and diseased central nervous system. *Nat. Immunol.* **24**, 393–407 (2023). 36759712 10.1038/s41590-022-01415-8

[10] Kierdorf, K., Masuda, T., Jordão, M. J. C. & Prinz, M. Macrophages at CNS interfaces: ontogeny and function in health and disease. *Nat. Rev. Neurosci.* **20**, 547–562 (2019). 31358892 10.1038/s41583-019-0201-x

[11] Prinz, M., Masuda, T., Wheeler, M. A. & Quintana, F. J. Microglia and central nervous system-associated macrophages-from origin to disease modulation. *Annu. Rev. Immunol.* **39**, 251–277 (2021). 33556248 10.1146/annurev-immunol-093019-110159 PMC8085109

[12] Prinz, M., Jung, S. & Priller, J. Microglia biology: one century of evolving concepts. *Cell* **179**, 292–311 (2019). 31585077 10.1016/j.cell.2019.08.053

[13] Paolicelli, R. C. et al. Synaptic pruning by microglia is necessary for normal brain development. *Science* **333**, 1456–1458 (2011). 21778362 10.1126/science.1202529

[14] Shigemoto-Mogami, Y., Hoshikawa, K., Goldman, J. E., Sekino, Y. & Sato, K. Microglia enhance neurogenesis and oligodendrogenesis in the early postnatal subventricular zone. *J. Neurosci.* **34**, 2231–2243 (2014). 24501362 10.1523/JNEUROSCI.1619-13.2014 PMC3913870

[15] Nimmerjahn, A., Kirchhoff, F. & Helmchen, F. Resting microglial cells are highly dynamic surveillants of brain parenchyma in vivo. *Science* **308**, 1314–1318 (2005). 15831717 10.1126/science.1110647

[16] Kierdorf, K. et al. Microglia emerge from erythromyeloid precursors via Pu.1- and Irf8-dependent pathways. *Nat. Neurosci.* **16**, 273–280 (2013). 23334579 10.1038/nn.3318

[17] Schulz, C. et al. A lineage of myeloid cells independent of Myb and hematopoietic stem cells. *Science* **336**, 86–90 (2012). 22442384 10.1126/science.1219179

[18] Bruttger, J. et al. Genetic cell ablation reveals clusters of local self-renewing microglia in the mammalian central nervous system. *Immunity* **43**, 92–106 (2015). 26163371 10.1016/j.immuni.2015.06.012

[19] Tay et al. A new fate mapping system reveals context-dependent random or clonal expansion of microglia. *Nat. Neurosci.* **20**, 793–803 (2017). 28414331 10.1038/nn.4547

[20] Huang, Y. et al. Repopulated microglia are solely derived from the proliferation of residual microglia after acute depletion. *Nat. Neurosci.* **21**, 530–540 (2018). 29472620 10.1038/s41593-018-0090-8

[21] Ingiosi, A. M., Opp, M. R. & Krueger, J. M. Sleep and immune function: glial contributions and consequences of aging. *Curr. Opin. Neurobiol.* **23**, 806–811 (2013). 23452941 10.1016/j.conb.2013.02.003 PMC3695049

[22] Imeri, L. & Opp, M. R. How (and why) the immune system makes us sleep. *Nat. Rev. Neurosci.* **10**, 199–210 (2009). 19209176 10.1038/nrn2576 PMC2839418

[23] Tuan, L. H. & Lee, L. J. Microglia-mediated synaptic pruning is impaired in sleep-deprived adolescent mice. *Neurobiol. Dis.* **130**, 104517 (2019). 31229687 10.1016/j.nbd.2019.104517

[24] Gentry, N. W. et al. Microglia are involved in the protection of memories formed during sleep deprivation. *Neurobiol. Sleep. Circadian Rhythms* **12**, 100073 (2022). 35028489 10.1016/j.nbscr.2021.100073 PMC8741522

[25] Bellesi, M. et al. Sleep loss promotes astrocytic phagocytosis and microglial activation in mouse cerebral cortex. *J. Neurosci.* **37**, 5263–5273 (2017). 28539349 10.1523/JNEUROSCI.3981-16.2017 PMC5456108

[26] Gu, X. et al. (2023). Imaging microglia surveillance during sleep-wake cycles in freely behaving mice. Elife *12*. 10.7554/eLife.86749. 10.7554/eLife.86749 PMC10746140 38132088

[27] Rowe, R. K. et al. (2022). Microglia are necessary to regulate sleep after an immune challenge. *Biology* 11. 10.3390/biology11081241. 10.3390/biology11081241 PMC9405260 36009868

[28] Picard, K. et al. Microglial homeostasis disruption modulates non-rapid eye movement sleep duration and neuronal activity in adult female mice. *Brain Behav. Immun.* **107**, 153–164 (2023). 36202169 10.1016/j.bbi.2022.09.016

[29] Corsi, G. et al. Microglia modulate hippocampal synaptic transmission and sleep duration along the light/dark cycle. *Glia* **70**, 89–105 (2022). 34487590 10.1002/glia.24090 PMC9291950

[30] Miyanishi, K. et al. Microglia modulate sleep/wakefulness under baseline conditions and under acute social defeat stress in adult mice. *Neurosci. Res* **202**, 8–19 (2024). 38029860 10.1016/j.neures.2023.11.010

[31] Giordano, K. R., Green, T. R. F., Opp, M. R. & Rowe, R. K. Microglial depletion and repopulation differentially modulate sleep and inflammation in a mouse model of traumatic brain injury. *Neurobiol. Sleep. Circadian Rhythms* **18**, 100115 (2025). 40703574 10.1016/j.nbscr.2025.100115 PMC12282821

[32] Liu, H. et al. Microglia modulate stable wakefulness via the thalamic reticular nucleus in mice. *Nat. Commun.* **12**, 4646 (2021). 34330901 10.1038/s41467-021-24915-x PMC8324895

[33] Ma, C. et al. Microglia regulate sleep through calcium-dependent modulation of norepinephrine transmission. *Nat. Neurosci.* **27**, 249–258 (2024). 38238430 10.1038/s41593-023-01548-5 PMC10849959

[34] Pinto, M. J. et al. Microglial TNFα orchestrates protein phosphorylation in the cortex during the sleep period and controls homeostatic sleep. *Embo J.* **42**, e111485 (2023). 36385434 10.15252/embj.2022111485 PMC9811617

[35] Dagher, N. N. et al. Colony-stimulating factor 1 receptor inhibition prevents microglial plaque association and improves cognition in 3xTg-AD mice. *J. Neuroinflammation* **12**, 139 (2015). 26232154 10.1186/s12974-015-0366-9 PMC4522109

[36] Van Hove, H. et al. A single-cell atlas of mouse brain macrophages reveals unique transcriptional identities shaped by ontogeny and tissue environment. *Nat. Neurosci.* **22**, 1021–1035 (2019). 31061494 10.1038/s41593-019-0393-4

[37] Bandarabadi, M. et al. A role for spindles in the onset of rapid eye movement sleep. *Nat. Commun.* **11**, 5247 (2020). 33067436 10.1038/s41467-020-19076-2 PMC7567828

[38] Bandarabadi, M. et al. Dynamic modulation of theta-gamma coupling during rapid eye movement sleep. *Sleep* **42**, 1–11 (2019). 10.1093/sleep/zsz182 31410477

[39] Vassalli, A. & Franken, P. Hypocretin (orexin) is critical in sustaining theta/gamma-rich waking behaviors that drive sleep need. *Proc. Natl. Acad. Sci. USA* **114**, E5464–e5473 (2017). 28630298 10.1073/pnas.1700983114 PMC5502606

[40] Endo, T. et al. Selective REM sleep deprivation in humans: effects on sleep and sleep EEG. *Am. J. Physiol.* **274**, R1186–R1194 (1998). 9575987 10.1152/ajpregu.1998.274.4.R1186

[41] Tononi, G. & Cirelli, C. Sleep and synaptic homeostasis: a hypothesis. *Brain Res. Bull.* **62**, 143–150 (2003). 14638388 10.1016/j.brainresbull.2003.09.004

[42] Hristovska, I. et al. Sleep decreases neuronal activity control of microglial dynamics in mice. *Nat. Commun.* **13**, 6273 (2022). 36271013 10.1038/s41467-022-34035-9 PMC9586953

[43] Kon, K. et al. (2023). Cortical parvalbumin neurons are responsible for homeostatic sleep rebound through CaMKII activation. bioRxiv, 2023.2004.2029.537929. 10.1101/2023.04.29.537929. 10.1038/s41467-024-50168-5 PMC11258272 39025867

[44] Bouhours, B., Gjoni, E., Kochubey, O. & Schneggenburger, R. Synaptotagmin2 (Syt2) drives fast release redundantly with Syt1 at the output synapses of parvalbumin-expressing inhibitory neurons. *J. Neurosci.* **37**, 4604–4617 (2017). 28363983 10.1523/JNEUROSCI.3736-16.2017 PMC6596661

[45] Morairty, S. R. et al. A role for cortical nNOS/NK1 neurons in coupling homeostatic sleep drive to EEG slow wave activity. *Proc. Natl. Acad. Sci. USA* **110**, 20272–20277 (2013). 24191004 10.1073/pnas.1314762110 PMC3864296

[46] Cirelli, C., Huber, R., Gopalakrishnan, A., Southard, T. L. & Tononi, G. Locus ceruleus control of slow-wave homeostasis. *J. Neurosci.* **25**, 4503–4511 (2005). 15872097 10.1523/JNEUROSCI.4845-04.2005 PMC6725032

[47] Willis, E. F. et al. Repopulating microglia promote brain repair in an IL-6-Dependent Manner. *Cell* **180**, 833–846.e816 (2020). 32142677 10.1016/j.cell.2020.02.013

[48] Zhang, Y. et al. Repopulating retinal microglia restore endogenous organization and function under CX3CL1-CX3CR1 regulation. *Sci. Adv.* **4**, eaap8492 (2018). 29750189 10.1126/sciadv.aap8492 PMC5943055

[49] Favuzzi, E. et al. GABA-receptive microglia selectively sculpt developing inhibitory circuits. *Cell* **184**, 4048–4063.e4032 (2021). 34233165 10.1016/j.cell.2021.06.018 PMC9122259

[50] Wilton, D. K., Dissing-Olesen, L. & Stevens, B. Neuron-glia signaling in synapse elimination. *Annu Rev. Neurosci.* **42**, 107–127 (2019). 31283900 10.1146/annurev-neuro-070918-050306

[51] Badimon, A. et al. Negative feedback control of neuronal activity by microglia. *Nature* **586**, 417–423 (2020). 32999463 10.1038/s41586-020-2777-8 PMC7577179

[52] Guillaumin, M. C. C. et al. (2023). Deficient synaptic neurotransmission results in a persistent sleep-like cortical activity across vigilance states in mice. bioRxiv, 2023.2005.2011.540034. 10.1101/2023.05.11.540034. 10.1016/j.cub.2025.02.053 40118064

[53] Seifinejad, A., Vassalli, A. & Tafti, M. Neurobiology of cataplexy. *Sleep. Med. Rev.* **60**, 101546 (2021). 34607185 10.1016/j.smrv.2021.101546

[54] Osorio-Forero, A. et al. Noradrenergic circuit control of non-REM sleep substates. *Curr. Biol.* **31**, 5009–5023.e5007 (2021). 34648731 10.1016/j.cub.2021.09.041

[55] Ma, C., et al. (2023). Microglia Regulate Sleep via Calcium-Dependent Modulation of Norepinephrine Transmission. bioRxiv, 2023.2007.2024.550176. 10.1101/2023.07.24.550176. 10.1038/s41593-023-01548-5 PMC10849959 38238430

[56] Stowell, R. D. et al. Noradrenergic signaling in the wakeful state inhibits microglial surveillance and synaptic plasticity in the mouse visual cortex. *Nat. Neurosci.* **22**, 1782–1792 (2019). 31636451 10.1038/s41593-019-0514-0 PMC6875777

[57] Liu, Y. U. et al. Neuronal network activity controls microglial process surveillance in awake mice via norepinephrine signaling. *Nat. Neurosci.* **22**, 1771–1781 (2019). 31636449 10.1038/s41593-019-0511-3 PMC6858573

[58] Maugard, M., Vigneron, P. A., Bolanos, J. P. & Bonvento, G. l-Serine links metabolism with neurotransmission. *Prog. Neurobiol.* **197**, 101896 (2021). 32798642 10.1016/j.pneurobio.2020.101896

[59] Vávrová, K., Hrabálek, A., Dolezal, P., Holas, T. & Zbytovská, J. L-Serine and glycine based ceramide analogues as transdermal permeation enhancers: polar head size and hydrogen bonding. *Bioorg. Med. Chem. Lett.* **13**, 2351–2353 (2003). 12824032 10.1016/s0960-894x(03)00409-8

[60] Masuda, T. et al. Novel Hexb-based tools for studying microglia in the CNS. *Nat. Immunol.* **21**, 802–815 (2020). 32541832 10.1038/s41590-020-0707-4

[61] Masuda, T. et al. Specification of CNS macrophage subsets occurs postnatally in defined niches. *Nature* **604**, 740–748 (2022). 35444273 10.1038/s41586-022-04596-2

[62] Lei, F. et al. CSF1R inhibition by a small-molecule inhibitor is not microglia specific; affecting hematopoiesis and the function of macrophages. *Proc. Natl. Acad. Sci. USA* **117**, 23336–23338 (2020). 32900927 10.1073/pnas.1922788117 PMC7519218

[63] Montilla, A. et al. Microglia and meningeal macrophages depletion delays the onset of experimental autoimmune encephalomyelitis. *Cell Death Dis.* **14**, 16 (2023). 36635255 10.1038/s41419-023-05551-3 PMC9835747

[64] Brennan, F. H. et al. Microglia promote maladaptive plasticity in autonomic circuitry after spinal cord injury in mice. *Sci. Transl. Med* **16**, eadi3259 (2024). 38865485 10.1126/scitranslmed.adi3259

[65] Green, K. N. & Hume, D. A. (2021). On the utility of CSF1R inhibitors. *Proc. Natl. Acad. Sci. USA* 118. 10.1073/pnas.2019695118. 10.1073/pnas.2019695118 PMC7848745 33446486

[66] Johnson, N. R. et al. CSF1R inhibitors induce a sex-specific resilient microglial phenotype and functional rescue in a tauopathy mouse model. *Nat. Commun.* **14**, 118 (2023). 36624100 10.1038/s41467-022-35753-w PMC9829908

[67] Behringer, S. et al. (2019). Targeted metabolic profiling of methionine cycle metabolites and redox thiol pools in mammalian plasma, cells and urine. *Metabolites* 9. 10.3390/metabo9100235. 10.3390/metabo9100235 PMC6836102 31635306

[68] Bravo, A. C. et al. Analysis of S-adenosylmethionine and S-adenosylhomocysteine: method optimisation and profiling in healthy adults upon short-term dietary intervention. *Metabolites* **12**, 373 (2022). 35629877 10.3390/metabo12050373 PMC9143066

[69] Hannibal, L. et al. Metabolic profiling in human fibroblasts enables subtype clustering in glycogen storage disease. *Front Endocrinol.* **11**, 579981 (2020). 10.3389/fendo.2020.579981 PMC7719825 33329388

[70] Maier, J. P. et al. Inhibition of metabotropic glutamate receptor III facilitates sensitization to alkylating chemotherapeutics in glioblastoma. *Cell Death Dis.* **12**, 723 (2021). 34290229 10.1038/s41419-021-03937-9 PMC8295384

[71] Moritz, L., Klotz, K., Grunert, S. C., Hannibal, L. & Spiekerkoetter, U. Metabolic phenotyping in phenylketonuria reveals disease clustering independently of metabolic control. *Mol. Genet Metab.* **138**, 107509 (2023). 36791482 10.1016/j.ymgme.2023.107509

[72] Franken, P., Malafosse, A. & Tafti, M. Genetic variation in EEG activity during sleep in inbred mice. *Am. J. Physiol.* **275**, R1127–R1137 (1998). 9756543 10.1152/ajpregu.1998.275.4.R1127

[73] Li, S., Franken, P. & Vassalli, A. Bidirectional and context-dependent changes in theta and gamma oscillatory brain activity in noradrenergic cell-specific Hypocretin/Orexin receptor 1-KO mice. *Sci. Rep.* **8**, 15474 (2018). 30341359 10.1038/s41598-018-33069-8 PMC6195537

[74] Tort, A. B. et al. Dynamic cross-frequency couplings of local field potential oscillations in rat striatum and hippocampus during performance of a T-maze task. *Proc. Natl. Acad. Sci. USA* **105**, 20517–20522 (2008). 19074268 10.1073/pnas.0810524105 PMC2629291

