# Supplementary material for: Contrasting contribution of resident and repopulated brain macrophages in sustaining sleep-wake circuitry
Source: Commun Biol. 2025 Sep 9;8:1339. doi: 10.1038/s42003-025-08781-7 (PMC12420833; doi:10.1038/s42003-025-08781-7)
Supplement: Supplementary file 1 — Supplementary Information [file 42003_2025_8781_MOESM1_ESM.pdf]

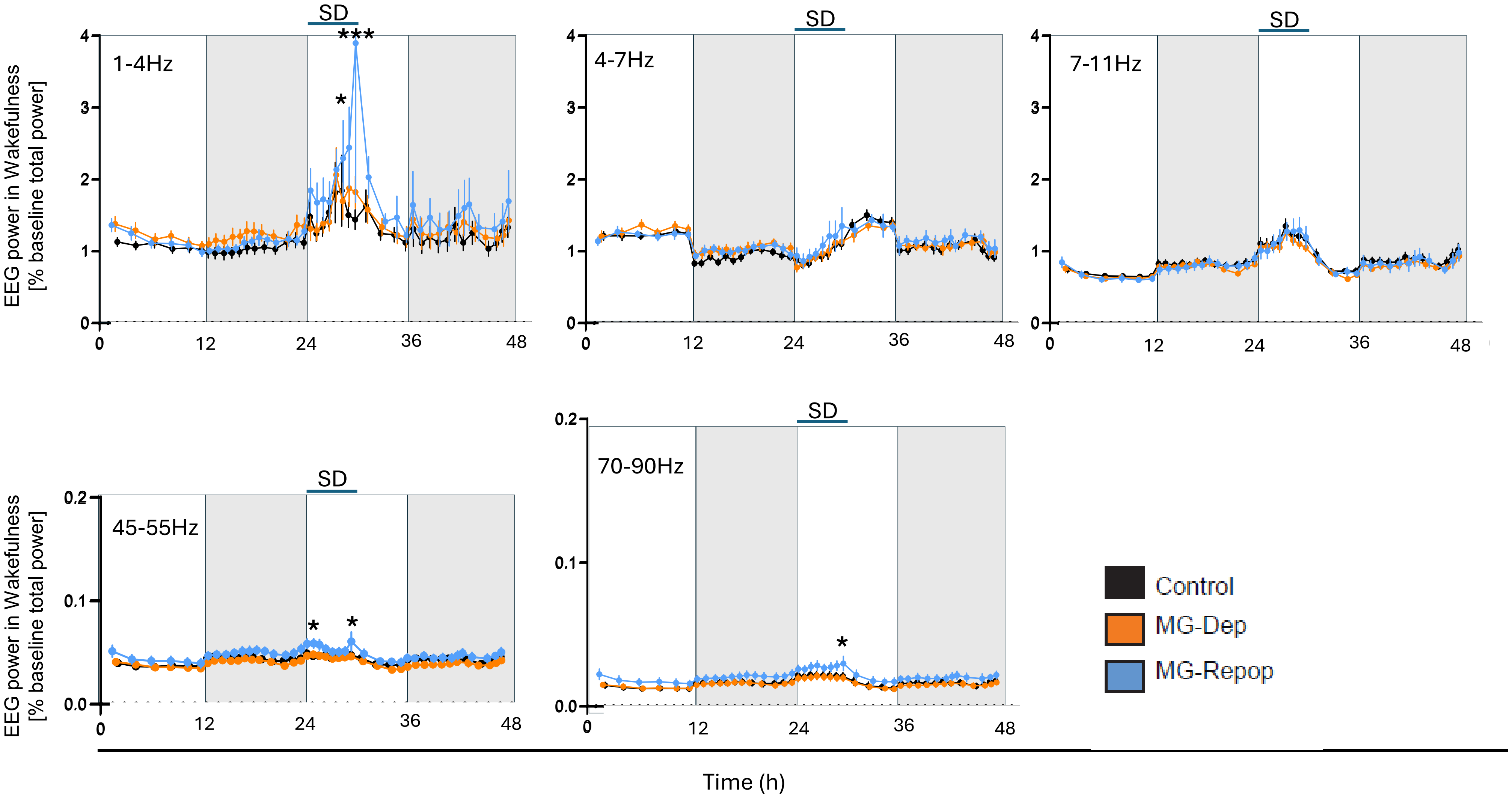

Supplementary figure 1. Time-course of waking EEG for different frequency band. Waking EEG delta (1-4Hz), Inter-delta/theta (4-7 Hz), Theta (7-11 Hz), slow-gamma (45-55 Hz) and fast-gamma (70-90 Hz) power dynamics during the 3-day recording (baseline days 1 and 2 are averaged) (2-way ANOVA, followed by Dunnett's test, *P < 0.05; **P < 0.01; ***P < 0.001). n = 9 for control and MG-Depleted conditions and n = 6 for MG-Repopulated condition.
