# Supplementary material for: Contrasting contribution of resident and repopulated brain macrophages in sustaining sleep-wake circuitry
Source: Commun Biol. 2025 Sep 9;8:1339. doi: 10.1038/s42003-025-08781-7 (PMC12420833; doi:10.1038/s42003-025-08781-7)
Supplement: Supplementary file 2 — Description of Additional Supplementary Materials [file 42003_2025_8781_MOESM2_ESM.pdf]

Description of Additional Supplementary Files

File name: Supplementary Data 1

Description: Source data
